# Exercise therapy for Stress-related mental disorder, a randomised controlled trial in primary care

**DOI:** 10.1186/1471-2296-12-76

**Published:** 2011-07-26

**Authors:** A Otto Quartero, Huib Burger, Marieke Donker, Niek J de Wit

**Affiliations:** 1 Julius Center for Health Sciences and Primary Care, University Medical Center Utrecht, Utrecht, the Netherlands; 2 Department of Epidemiology and Department of Psychiatry, University Medical Centre Groningen, Groningen, the Netherlands

**Keywords:** stress-related mental disorder, exercise therapy, mental health, general practice, occupational health

## Abstract

**Background:**

to investigate whether a structured physical exercise programme (PEP) improves the recovery of general health in patients suffering from Stress-related Mental Disorder (SMD).

**Method:**

Study design: randomised open trial in general practice. Patients from two regions in the Netherlands were included between September 2003 and December 2005, and followed up for 12 weeks.

Intervention: the patients were referred to a physical therapist for instruction in and monitoring of physical exercise of an intermediate intensity. Following the Dutch Guidelines for Healthy Physical Exercise, the patients were instructed to exercise at least five times a week, for at least 30 minutes per day.

Control group: usual care from the GP

**Outcome:**

*Primary: *improvement of general health after 6 weeks according to the 'general health' dimension of the Short-Form 36.

*Secondary: *total days off work, percentage that resumed work after 6 and 12 weeks, change in distress score and change in remaining SF36 dimensions after 6 and 12 weeks.

**Results:**

out of 102 randomised patients (mean age 43, 60 (59%) female), 70 (68%) completed the trial, of whom 31 were in the intervention group. After 6 weeks, the mean (SD) general health score was 54.6 (22.1) for the intervention group and 57.5 (19.2) for the controls. The corresponding effect size (Cohen's *d *with 95% confidence interval) from analysis of covariance was -0.06 (-0.41, 0.30) indicating no effect on general health. No significant effects of the intervention were detected for any secondary outcome parameter either.

**Conclusion:**

Notwithstanding the relatively high drop-out rate, our results suggest that referral to a physical therapist for structured physical exercise is not likely to be very effective in improving recovery from SMD.

**Trial registry:**

Current Controlled Trials ISRCTN15609105

## Background

Stress-related Mental Disorder (SMD) is a common problem in general practice. In the Diagnostic and Statistical Manual of Mental Disorders-IV SMD is partly but not exclusively covered by 'adjustment disorder', and in the International Classification of Diseases-10 by adjustment disorder (F43.2), neurasthenia (F48.0), to some extent burn-out (Z73.0) and work-related disorders (Z56.1-7). It is more generally known as a nervous breakdown or being 'overstressed' or 'overburdened'. These descriptions and definitions are interrelated, which is why the term SMD was introduced by Terluin [1,2]. SMD indicates relevant dimensions of psychopathology that are sub acute and related to stress: the common psychopathology often starts with a failure to cope with personal, social or occupational demands, and distress will follow. Depleting psychological resources often lead to sick leave, because the patient stops trying to cope and gives in. The mental stress usually develops in a professional employment environment, but negative personal circumstances and major life events can also contribute to it. On the other hand, coping skills, social support and personality factors also play a part. SMD is diagnosed when a patient experiences a significant impairment of personal, social or professional function. SMD causes a significant reduction in quality of life and induces substantial sick leave. In the Netherlands, the incidence of this condition is 12/1000/year, meaning one new patient every two weeks in an average general practice [3,4]. Most patients with this disorder (85%) are adults with a paid job [2]. The social implication of this condition is major: 50% of patients take sick leave even before the first visit to the GP. There is a risk of permanent functional impairment due to the development of anxiety disorder, somatisation or a depressive disorder. In the present situation, only 38% of patients have recovered after one month and 59% after 6 months [2]. For these reasons, SMD is a relevant health problem both in quantitative and qualitative terms. The treatment goal for patients with SMD is recovery of perceived health, with a focus on retrieving control at the cognitive and emotional levels, and on relieving distress. To achieve these goals, the General Practitioner (GP) generally offers supportive consultations in line with the professional recommendations of the Dutch College for General Practice [5]. Together, the GP and patient analyse the distressing factors and explore potential solutions ('care as usual') [6].

In these supportive consultations the GP advises relaxation and a break from stressful situations, encourages daily activities and suggests creating emotional distance from work. In due course, some patients will be referred to a social worker or a first-line psychologist.

However, there is little scientific evidence as to the effectiveness of therapies for SMD. There is no evidence for the effectiveness of pharmacological treatments. When the symptoms of anxiety or depression obstruct recovery, benzodiazepines or antidepressants may be required [6].

Terluin et al. published a systematic review of various therapies for patients with SMD. This review demonstrates that non-medical interventions of a short duration aimed at physical activity can have a positive effect on role functioning [6]. In patients with depressive disorders, physical exercise has been shown to have a positive effect on recovery and may prevent relapses [7,8]. Furthermore, in persons without clinically diagnosed depression physical activity improves mood, reduces anxiety and increases stress-resistance and self-reported health [9]. For the GP, the initiation and monitoring of physical activity in patients with SMD is probably less difficult than for other caregivers, as he or she has a good understanding of the patient's general health and social environment and is familiar with the local sports facilities. In addition, access to a GP for monitoring progress and development is easy and inexpensive. For the patient, increased physical activity stimulates health-improving behaviour that may contribute to an early recovery. However, the evidence for a beneficial effect of physical exercise in SMD is lacking.

Our hypothesis was that the referral to a structured physical exercise programme of patients with SMD would enhance their recovery within 6-12 weeks. We report the results of a primary care-based, randomised trial analysing the effectiveness of a structured programme of physical activity in improving the general health of patients with SMD.

## Methods

### Design

Randomised, open, general practice-based pragmatic clinical trial.

### Ethical aspects

The Medical Ethic Review Board of the University Medical Centre of Utrecht approved the study.

### Patients

Patients with SMD were selected in a stepwise manner from 23 participating primary care practices in two provinces in the middle and east of the Netherlands. GPs were invited to participate without any exclusion criteria. Selections were made from the GP's electronic files. Patients with SMD who were between 18 and 65 years were eligible. Exclusion criteria were active depression (i.e. receiving any form of therapy), anxiety disorder, drug addiction or other major psychiatric diagnoses on axis I of DSM-IV. There was no medical restriction for participation, nor were more active patients, as assessed by the physical therapist, excluded.

### Procedure

In the first phase, patients who visited the surgery within the last four weeks with a diagnosis in the mental health dimension (code P) of the International Classification of Primary Care (ICPC) were identified in the electronic medical files of the participating practices. An additional search was carried out on text words linked to SMD. Along with an introductory letter from their GP, these patients also received a screening list for SMD, which was specifically developed for this study. It comprised three questions: have you suffered from worrying, have you suffered from listlessness or have you suffered from tenseness in the past week? The answer categories were 'no' (code 0), 'sometimes' (code 1) or 'often' (code 2). The patients calculated their own scores; the screening test was considered positive if they had score > 3. In a pilot study score > 3 was demonstrated to rule in SMD with 85% sensitivity and 96% specificity (Terluin, personal communications). Patients with a positive score were eligible for the study and were invited in writing to participate. They received full information about the study and were asked to complete the Informed Consent forms. If they did not reply within two weeks they were approached by telephone.

The patients were randomised using a computer-generated list available at the trial unit of the University Medical Center Utrecht as soon as the informed consent forms were received. The treatment allocation was concealed as follows: the person who generated the randomisation list and reported the allocation codes to the researcher was not involved in determining patients' eligibility or any other aspect of the conduct of this study. He was instructed to assign patients in sequence. Furthermore, the fixed block size of four was unknown to the researchers.

### Intervention

The patients were randomised either to intervention or to care as usual. The patients in the intervention group were referred to cooperating physical therapists, who designed an individually structured physical exercise programme (PEP) based on the Dutch Standard for Healthy Exercise. This comprises moderate intensive exercise on at least five days a week for at least 30 minutes [10]. Moderate exercise is defined as between 50 and 85% of maximal individual exercise as estimated on the basis of age and gender. Examples of activities include walking, swimming or cycling; the national standard is based on international research and guidelines [11,12]. The therapist instructed the patient on the level of perceived exertion. The therapists were instructed to monitor the exercise programme with the patient for 12 weeks following a preset schedule. The patient visited the physical therapist eight times: twice in the first week, once a week in weeks 2-6 and in the remaining weeks once or twice per three weeks. We obtained no reports from the therapists as to how the exercise was done, nor about what the content of the monitoring visits was. The therapists were instructed in writing and by telephone, but received no further training; nor were they supervised.

In the control group the patients received usual care from their GP, i.e. supportive and explorative consultations, with pharmacotherapy if indicated.

### Outcome

The primary outcome was the difference in change in general health 6 weeks after inclusion in the study. The secondary outcomes were total days off work, percentage that resumed work after 6 and 12 weeks, change in distress score and change in the dimensions of mental health, social health and role functioning after 6 and 12 weeks

### Measurements

General health, social functioning and mental health are most affected in SMD [1].

For the primary outcome we selected the general health dimension of the SF-36. The general health dimension comes close to what GPs consider 'health in general terms' [13,14].

The SF-36 is a validated instrument that measures eight dimensions of quality of life based on self-report. We used the standard four-week recall version. The SF-36 scores from 0 to 100, with 100 indicating the optimal health dimension. It is a well-known, internationally accepted instrument to measure quality of life; the general health dimension comes close to what GPs consider 'health in general terms' [13,14].

We used the four-dimensional symptom questionnaire (4DSQ) to differentiate distress in SMD patients from depression, anxiety and somatisation. The 4DSQ comprises 50 questions on a four-point Likert scale. It was developed and validated in Dutch general practice and measures four dimensions of psychopathology: distress, depression, anxiety and somatisation. Distress is the dimension that is expected to be abnormal in SMD patients. The scale is self-reported and can be used for research as well as for clinical practice in patients from 15 years of age [1]. All questionnaires were completed on paper by the patients and returned by post.

### Sample size

In view of the substantial burden on the patient of our intervention we decided that only moderate to large effect sizes (Cohen's *d *in the range 0.5 up to 1.0) would be of clinical interest [15]. Moderate to large effect sizes for general health equate to the effect of recovering from depression or a sleeping disorder [16]. Our sample size calculation was therefore based on an effect size of 0.75 (midpoint of 0.5 and 1.0) for the primary outcome, i.e. the SF-36 general health dimension at week 6. The standard deviation (SD) of this dimension of the SF-36 in the age range of 41 to 60 years was 20.6 according to data from a large general population sample [17]. With this value for the SD, an effect size of 0.75 corresponds to a 15-point difference between groups. To demonstrate such a difference with 90% power, an alpha value of 0.05 and equal allocation rates, 41 patients would be needed in each group. Assuming a 15% loss to follow-up rate, we had to include 48 patients per group. This rate was based on our experience.

We rated the distress of participants by using the distress score at baseline and at 6 and 12 weeks.

We counted the percentage of weekly self-reported hours off duty in both groups to estimate the economic effect of the intervention on the number of sick leave days.

### Statistical analysis

The control and intervention groups were first compared for baseline characteristics. To prevent bias from missing values on the 4DSQ and the SF 36 at baseline (N = 102), 6 weeks (N = 87) and 12 weeks (N = 70), we performed a single imputation based on multiple linear regression. We then analysed the effectiveness of the physical activation programme at 6 weeks and 12 weeks on four dimensions of quality of life and four dimensions of psychopathology using analysis of covariance. This method is less sensitive to regression to the mean and has more statistical power than analysis of the difference of change in the outcome variable [16]. In an additional analysis we adjusted for differences in baseline characteristics. For each analysis we calculated the effect size (Cohen's *d*). A level of significance (alpha) of 0.05 was used.

## Results

### Patients

**Figure 1:**
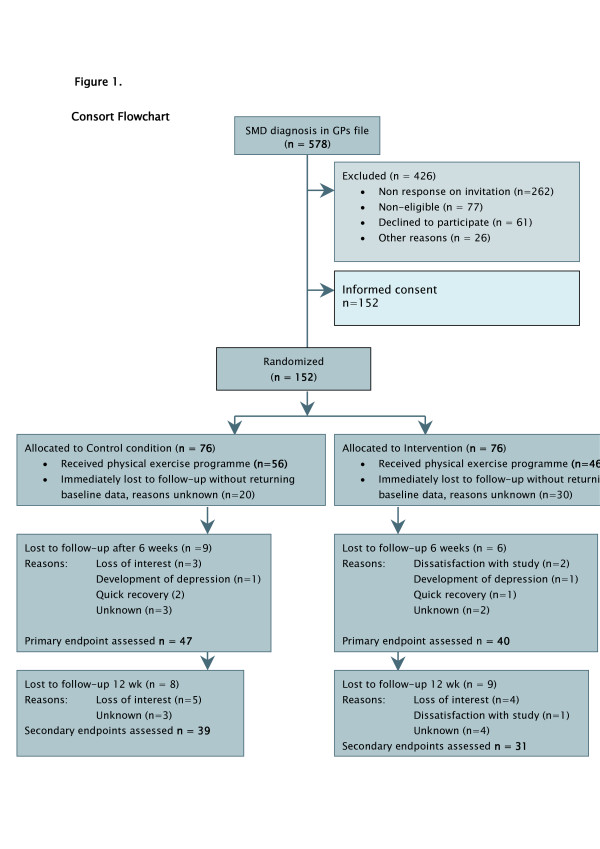
**Consort Flow Chart**. SMD = Stress-related Mental Disorder; GP = general practitioner.

During the research period, 578 patients with a clinical suspicion of SMD from the participating practices were identified and invited to participate (see flow chart, Figure 1). Of these, 262 patients (45%) did not respond. Of the 316 patients (55%) who did return the screenings lists, 164 (28% of total) did not meet the inclusion criteria. Informed consent was given by 152 patients (27% of those approached). These patients were randomised and received the baseline questionnaires.

Of the 152 patients randomised, only 102 (67%) returned the forms with baseline data and entered the trial, 56 (37% of those randomised) in the control group and 46 (30% of those randomised) in the intervention group. The remaining 50 patients (33%) immediately dropped out after randomisation, did not return baseline data and abstained from participation.

Of the 102 patients who actually entered the trial, 87 (85%) reached the primary endpoint at 6 weeks, and 70 (68%) reached the secondary endpoint at 12 weeks. Reasons for drop-out after entry were various: development of depression (N = 2), dissatisfaction with the physical exercise programme (N = 2), loss of interest in the study (N = 3), quick recovery from the SMD (N = 3) and reason unknown (N = 5)

## Results

The demographic characteristics of the patients in the control group did not differ from those of the patients in the intervention group (table 1). The baseline scores for quality of life and 4DSQ scores for depression and distress in the two groups were similar; for anxiety and somatisation the 4DSQ levels were slightly higher in the intervention group (table 1).

**Table 1:** Baseline characteristics

	PEP (N = 46)	Care as usual (N = 56)	p value
**Demographics**			
**Age in years (SD)**	43.7 (9.6)	42.5 (10.4)	0.56
**% Female**	61	57	0.70
**Psychopathology (SD)**			
**4DSQ Distress dimension**	21.7 (6.1)	19.7 (7.3)	0.15
**4DSQ Depression dimension**	3.7 (3.1)	3.0 (3.0)	0.26
**4DSQ Anxiety dimension**	6.6 (5.4)	4.3 (4.5)	0.02
**4DSQ Somatisation dimension**	13.3 (6.5)	10.8 (5.0)	0.03
**Quality of life (SD)**			
**SF-36 score general health**	49.9 (20.6)	53.7 (20.5)	0.35
**SF-36 Mental Health**	39.8 (18.4)	45.0 (17.7)	0.14
**SF-36 Social Functioning**	47.4 (28.5)	48.3 (25.2)	0.86
**SF-36 Role Functioning**	21.0 (36.8)	23.4 (35.9)	0.74

PEP = Physical Exercise Programme; 4DSQ = 4-dimensional symptom questionnaire; SF-36 = short form 36-item version; SD = standard deviation.

### Outcome

After 6 weeks there was no difference between the two groups in the change of the score on the SF 36 dimension general health (table 2, 3, 4). In addition, the change in mental health, social health and role functioning did not differ between the control and the intervention group. The percentage of hours of work (table 5) and the distress score (table 6) at 6 weeks was similar in the two groups.

**Table 2:** Effect of a Physical exercise programme (PEP) vs Care as usual (CAU) on general health and mental health (SF-36 score) at 6 and 12 weeks vs Baseline, using Analysis of covariance (ANCOVA) *

	General Health				Mental health			
	PEP	CAU	Difference (95% BI)	Effect size (95% BI)	PEP	CAU	Difference (95% BI)	Effect size (95% BI)
**Baseline**	49.9 (20.6)	53.7 (20.5)			39.8 (18.4)	45.0 (17.7)		
**6 weeks**	54.6 (22.1)	57.5 (19.2)			56.3 (16.1)	56.6 (14.5)		
**ANCOVA**			-1.2 (-8.5,6.2)	-0.06 (-0.41, 0.30)			1.2 (-4.5,6.9)	0.07 (-0.25, 0.38)
**12 weeks**	52.9 (22.2)	61.0 (20.7)			53.9 (18.9)	60.7 (16.1)		
**ANCOVA**			-5.7 (-12.7,1.2)	-0.26 (-0.62, 0.06)			-5.3 (-12.0,1.4)	-0.29 (-0.66, 0.08)

* ANCOVA analysis of covariance; Values are means (SD) unless indicated otherwise. 95% BI 95%-confidence interval; Effect size is Cohen's *d*; PEP = physical exercise programme; CAU = care as usual.

**Table 3:** Effect of a Physical exercise programme (PEP) vs Care as usual (CAU) on social functioning and role functioning (SF 36 score) at 6 and 12 weeks vs Baseline, using Analysis of co-variance (ANCOVA) *

	Social functioning				Role functioning			
	PEP	CAU	Difference (95% BI)	Effect size (95% BI)	PEP	CAU	Difference (95% BI)	Effect size (95% BI)
**Baseline**	47.4 (28.5)	48.3 (25.2)			21.0 (36.8)	23.4 (35.9)		
**6 weeks**	57.8 (23.2)	63.4 (21.4)			36.7 (42.7)	49.8 (44.6)		
**ANCOVA**			-5.4 (-13.8,3.1)	-0.02 (-0.52, 0.12)			-12.8 (-30.1,4.5)	-0.34(-0.80, 0.12)
**12 weeks**	60.1 (27.1)	69.9 (22.2)			37.4 (41.7)	62.3 (43.0)		
**ANCOVA**			-9.5 (-18.6,-0.5)	-0.36 (-0.70, -0.02)			-24.4 (-41.1,-7.8)	-0.65 (-1.09, -0.21)

* ANCOVA analysis of covariance; Values are means (SD) unless indicated otherwise. 95% BI 95%-confidence interval; Effect size is Cohen's *d*; PEP = physical exercise programme; CAU = care as usual.

**Table 4:** Effect of a Physical exercise programme (PEP) vs Care as usual (CAU) on general health, mental health and social functioning (SF-36) at 6 and 12 weeks, after correction for age, gender, and all 4DSQ baseline scores

	General health 6 wk	General health 12 wk	Mental health 6 wk	Mental health 12 wk	Social functioning 6 wk	Social functioning 12 wk	Role functioning 6 wk	Role functioning 12 wk
**p-value**	0.87	0.14	0.29	0.15	0.56	0.15	0.39	0.02

**Table 5:** Sick leave in intervention and control groups at 6 and 12 weeks

	Mean absence from work, in % of hours work		p-value for difference
	PEP	CAU	
**6 weeks** **N = 65**	30.0	29.7	0.98
**12 weeks** **N = 60**	31.3	25.7	0.59

**Table 6:** Mean (SD) distress score in intervention and control groups at 6 and 12 weeks

	Mean distress score		p-value for difference
	PEP	CAU	
**Baseline** **N = 96**	21.4 (6.1)	18.8 (7.3)	0.08
**6 weeks** **N = 87**	15.2 (7.6)	15.0 (7.4)	0.89
**12 weeks** **N = 80**	13.7 (7.8)	13.8 (8.0)	0.97

PEP = physical exercise programme; CAU = care as usual.

At 12 weeks we observed a significantly higher score in the control group for social functioning and for role functioning (score difference -9.5, 95% CI -18.6; -0.5 and -24.4, 95%CI -41.1,-7.8 respectively (table 2)).

We did not detect any differences in change on general health, mental health score (table 2), hours off work (table 5) or distress score (table 6).

Finally, in a multivariable analysis, after correction for baseline differences in distress score, SF 36 and 4DSQ scores, we could not identify statistical differences between the two groups in the scores on the general health, mental health, social functioning or role functioning dimensions of the SF36 score, neither at 6 weeks nor at 12 weeks, except for a small negative effect of the intervention on role functioning after 12 weeks (table 4).

### Drop-outs

Of 578 eligible patients, only 152 gave informed consent and were randomised. Additionally, we had a high number of patients dropping out immediately after randomisation (33%, 50 out of 152 patients). Only 87 of the 102 remaining patients (57% of those randomised) reached the primary endpoint.

Those who had dropped out by week 12 (N = 32) were remarkably similar to the completers as to baseline characteristics, except for the role functioning dimension of the SF-36 which showed a slightly lower score for the drop-outs compared with completers (15.8 vs. 25.0). This regarded the control group (28.8 vs. 11.11) more than the intervention group (22.0 vs. 20.6). However, none of the baseline differences were statistically significant. Unfortunately, we were unable to compare baseline data between participants and those 50 patients who immediately dropped out after randomisation because they failed to return the pertaining questionnaires. In addition, they did not provide reasons for dropout.

## Discussion

We found no difference in change in general health perception between patients who were referred for a structured physical exercise programme and those receiving care as usual, neither after 6 weeks nor after 12 weeks. Mental health perception and sick leave patterns in the two groups were similar. A slightly unfavourable effect of exercise on social functioning and on role functioning was observed in this patient group.

To our knowledge this is the first randomised clinical trial investigating the contribution of physical exercise to the recovery from stress-related mental disorder.

Although several studies have suggested that physical exercise may be beneficial to the speed of recovery of patients with depression and chronic fatigue disorder, we could not confirm a positive effect of a structured physical exercise programme on the perceived quality of life of patients with stress-related mental disorder [8,18,19].

However, we faced problems conducting the study, problems that were in our view directly related to the population under study and the logistics of the trial. We assume the high drop-out rate was due to the instability of the patients and thus a direct consequence of their stress-related disorder. Many patients who indicated interest in the study abandoned the protocol as soon as they were confronted with the consequences. Yet this rate was similar in both arms of the study. It is inherent to the disease that patients with stress-related disorder feel tired and lack energy, even though rationally they think they should exercise. In addition, we faced a logistical problem, as it turned out that the health insurance company of some of the patients was not willing to fund the physical exercise programme, despite confirming participation at an earlier stage. Some patients who would have had to pay for the PEP themselves declined further participation.

In the 'care as usual' group, patients may have dropped out because they felt disappointed to be randomised away from the intervention that initially attracted their attention. To examine any possible effect on the results of dropout during the intervention phase, we performed an analysis of those who completed the study up to week 6 and of those up to week 12. The results were essentially the same. As to the primary study outcome, for example, the difference in the change in general health after 6 weeks was -2.9 (95%CI: -9.7; 3.7, P-value 0,38) when we restricted our analyses to those participants who completed the study up to week 6. This difference at week 12 was -0.5 (95%CI: -6.0; 7.0, P-value 0,88) when restricted to those who were in the study up to week 12. These patterns were not any different when we adjusted for age, gender and 4-DSQ baseline scores.

Although the number of patients lost in the two groups during the follow-up was similar (16% vs13% at 6 weeks and 30 vs 37% at 12 weeks in intervention and control group), it was the excessive dropout during the immediate post-randomisation phase (50/152, 33%) that was most remarkable. Out of these patients, 30 had been randomized to the intervention and 20 to the care as usual group. At that time, the intervention had not been implemented. Therefore, dropout is in our view unlikely to be the result of the intervention in itself. However, self-selection at this stage may have occurred as a result of the mere anticipation of the exercise programme or, alternatively, of standard care, and would thus have been an expression of disappointment. The extent and impact of this potential selection bias is unknown and we were unable to compare the baseline characteristics of these early dropouts with participants because they did not return the questionnaires. Neither did they provide reasons for dropping out. The substantial losses to follow-up represent a major limitation of our study. Nevertheless, we consider it unlikely that bias due to dropout can explain our results completely.

The choice of our intervention may be questioned. We recruited our patients from 23 different general practices and at least as many physical therapists were involved in monitoring the PEP. It can be argued that the frequency, duration, intensity and nature of the exercise programme were too diverse to show a possible effect. However, it is not clear whether these variables have any influence on the recovery from SMD; indeed, the neurobiological action of a PEP is altogether unclear. It is believed that in depressive disorders a PEP works by stimulating the production of neuropeptides and by neuroadaptation [20]. In our study we pragmatically chose a PEP that could be adjusted to the patient's personal preferences, as long as the intensity was within the set limits of 50-85% of personal maximum, the duration was at least 30 min and the frequency was at least five times a week. Given the results of our study, we recommend a more uniform intervention in order to rule out any dilution from the effect of the type of exercise.

Due to the nature of the randomised interventions, neither the researcher nor the patients could be blinded. This may have caused some bias inherent to the use of self-report questionnaires.

## Conclusions

As we did not detect differences in improvement in any of the primary and secondary outcomes, and as we have evidence that dropout was not selective, we conclude from our findings that the effectiveness of referral to a physical therapist for structured physical exercise may be seriously questioned.

## Competing interests

The authors declare that they have no competing interests.

## Authors' contributions

MD was involved in data collection and drafting the article. AOQ was involved in design, supervision, and drafting the article; HB was involved in design, data analysis and drafting the article; NdW was involved in design and drafting the article. All authors agree to the final version of the paper.

## Pre-publication history

The pre-publication history for this paper can be accessed here:

http://www.biomedcentral.com/1471-2296/12/76/prepub
